# Haplotype and linkage disequilibrium of *TP53-WRAP53* locus in Iranian-Azeri women with breast cancer

**DOI:** 10.1371/journal.pone.0220727

**Published:** 2019-08-06

**Authors:** Nasser Pouladi, Sepehr Abdolahi, Davoud Farajzadeh, Mohammad Ali Hosseinpour Feizi

**Affiliations:** 1 Department of Biology, Faculty of Basic Sciences, Azarbaijan Shahid Madani University, Tabriz, Iran; 2 Department of Molecular Biology and Cancer Research, Azarbaijan Shahid Madani University, Tabriz, Iran; 3 Department of Animal Biology, Faculty of Natural Science, Tabriz University, Tabriz, Iran; Universitat des Saarlandes, GERMANY

## Abstract

Among the cancer susceptibility genes, *TP53* is one of the crucial genes involved in cell cycle regulations and, therefore, it greatly affects breast cancer initiation and progression. In addition, *WRAP53*—a natural antisense transcript—regulates *TP53* transcription and, as a protein, modulates the normal cell cycle, which results in breast cancer susceptibility. In this study, we aimed to analyze a haplotype comprising four SNPs, including rs1042522, rs17878362, rs2287499, and rs2287498, which are located at 5′ regions of the *TP53* and *WRAP53* genes, in 118 patients and 110 healthy controls of the Iranian-Azeri population. *In silico* studies were conducted using the SIFT, Polyphen2, Fanthmm, RNAsnp, and SNP&GO online servers. Linkage disequilibrium (LD) and D′ for each combination of the markers were calculated via the Haploview program. Our results showed that the GA_1_CC haplotype was the most frequent in the studied population. Additionally, no significant LD between any pairwise haplotypes was observed. The GA_1_CC and CA_2_GC haplotypes were significantly associated with breast cancer susceptibility. Moreover, the *in silico* analysis revealed the negative effects of rs2287499 and rs1042522 on *WRAP53* and P53, respectively. In conclusion, the CA_1_GC haplotype was strongly identified as a breast cancer risk factor, and the GA_1_CC haplotype was assumed to be a protective factor against breast cancer risk. Hence, these markers may potentially be used as molecular prognostic and predictive biomarkers for breast cancer.

## Introduction

According to the International Agency of Research on Cancer (IARC) in 2018, breast cancer is the second cause of death in both genders, and is the leading cause of mortality in women worldwide [1]. Various factors can contribute to breast cancer risk, including body mass index, breastfeeding, age at menarche, first birth, and menopause [2]. In addition, genetic and hereditary factors account for a considerable portion of diagnosed cases [3] such as family history of breast or ovarian cancer and inherited mutations in breast cancer susceptibility genes of which the *TP53* gene has a crucial role in breast cancer risk [3,4]. In response to cellular stresses, the p53 protein induces the expression of genes involved in cell cycle, senescence, apoptosis, and DNA repair regarding the context and extent of the stress [5]. As a tumor suppressor and tetrameric protein, any mutations in its coding region may affect its capability of establishing a functional protein [6]. *TP53* mutations are found in over 50% of all types of cancers [7]. It is located on chromosome 17p13.1 composed of 11 exons, 10 of which are coding and the first one is a noncoding exon [8]. A complex regulatory mechanism exists to maintain its functionality in which at the protein level, Mdm2 and Mdm4 [9], and at the RNA level, HuR, L26 RPL26, Wig-1 [10], miR125a [11], and *WRAP53*α [12] are known to be involved. On the opposite strand of and overlapped with *TP53*, another gene, called *WRAP53*, encodes three different products as a result of its three alternative exons [13]. *WRAP53*α, a noncoding transcript, regulates p53 mRNA through binding to its 5′ untranslated region (UTR) and thus stabilizes it. Although the mechanisms involved are still unclear, this interaction presumably leads to masking or exposing the p53 mRNA sequence to destabilizing or stabilizing elements, respectively [14]. *WRAP53*β (also known as TCAB1), a coding transcript, plays a key role in RNP biogenesis and telomerase trafficking to the Cajal body [13,15] and is also needed for p53-dependent apoptosis upon DNA damages and p53-indepentent double-strand break repair [16,17]. No specific function has been proposed for *WRAP53*γ up until now.

Single Nucleotide Polymorphisms (SNPs) may alter gene regulation and structure and result in aberrant RNA or protein dysfunction. Although cancer-associated SNPs in determinant genes can increase the risk of cancer, as shown already for *TP53* and *WRAP53* in breast cancer susceptibility [18,19,20], it is necessary to define haplotype blocks to investigate the role of neighboring SNPs in cancer risk and calculate the linkage disequilibrium (LD) [21]. LD is the association between two nearby markers (e.g., SNPs), that result from common inheritance and are influenced by population size and mutation age. Further, a haplotype is a set of co-inherited SNPs that pass down through generations as a single unit [22]. Many studies have investigated different haplotypes’ role in breast cancer susceptibility to elucidate the effects of ancestral SNPs in a given population [22,23,24]. Our goal in this study was to conduct a haplotype-based association analysis at the *TP53*-*WRAP53* locus in breast cancer. To do so, we analyzed the association between four SNPs, including rs1042522, rs17878362, rs2287499, and rs2287498, which refer to R72P substitution in exon 4 of *TP53* [25], a 16 bp duplication in intron 3 of *TP53* [26], a R68G substitution in the first exon of *WRAP53* [27], and a *WRAP53* Ex2+19 C>T polymorphism, respectively, in Iranian-Azeri women to validate the question of “whether this haplotype has a potential to be utilized as a prognostic biomarker for breast cancer or not”.

## Materials and methods

### Subjects

The study population comprised of 118 breast cancer patients and 110 healthy controls with no history of cancer. All subjects were selected from among the Iranian-Azeri population. Full written consents for all subjects were then obtained and approved by the Ethics Committee of Tabriz University of Medical Sciences research center (ethical approval code: 27997N1R2). This study protocol follows the ethical guidelines of the 1975 Declaration of Helsinki.

### SNPs selection and genotyping

The selected SNPs for this study consisted of five polymorphisms spanning 7,674–7,689 kb of chromosome 17 at the *TP53*-*WRAP53* locus, three of which (rs17880604, rs1042522, rs17878362) are located at 5′ region of the *TP53* gene and two of which (rs2287498, rs2287499) are located at 5′ region of the *WRAP53* gene.

In the *TP53* gene, rs17880604, rs1042522, and rs17878362 polymorphisms were genotyped by RFLP-PCR, ARMS-PCR, and PCR with silver staining methods, respectively, as previously described [25]. The genotype of rs2287499 polymorphism in the *WRAP53* gene was determined using SSCP-PCR as described by Bonab et al. [27]. The *WRAP53* rs2287498 polymorphism was genotyped by tetra-ARMS-PCR method. PCR-amplification was carried out in a total volume of 20 μl reaction mixture, including 2 μl PCR buffer (10X), 1.2 μl MgCl_2_ (50 μM), 1 μl of each external primers (10 μM), 0.3 μl of each internal primers, 0.62 μl dNTPs (10 μM), 13.35 μl sterile distilled H_2_O, 0.23 μl *Taq* DNA polymerase (5 unit μl^-1^) and 1 μl template DNA (20–25 ng), all were purchased from Takapouzist company, Iran. PCR was conducted in a thermal cycler (Sensoquest, GmbH, Germany) with the following cycling setting: an initial denaturation step for 10 minutes at 95°C, followed by 35 cycles of 30 seconds at 95°C for denaturation, 30 seconds at 58°C for primer annealing, 35 seconds at 72°C for extension, and also a final extension was carried out for 10 min at 72°C. The amplified products and a 50-bp DNA ladder, as molecular size marker, were loaded on 2% agarose gel wells, and then electrophoresis was conducted for 30 minutes at 8 V cm^-1^.

### Data and *in silico* analysis

To verify the Hardy-Weinberg equilibrium, P-value was set at 0.001. The frequency of polymorphisms and combinations of the three SNP markers were evaluated via the Haploview program v4.2 [28] based on P-value less than 0.05 as significant. A permutation test was also performed using this program to check the differences of haplotypes distribution between controls and breast cancer patients. In addition, calculating of Lewontin's standardized pairwise LD coefficient (D′) between each combination of the markers was done to determine the linkage disequilibrium. The *in silico* analysis was conducted to determine the deleterious and harmful effect of diseases, cancer-associated SNPs and SNP on RNA secondary structure using SIFT, Polyphen2, Fanthmm, RNAsnp, and SNP&GO online servers for three of the mentioned polymorphisms (e.g., rs2287498, rs2287499, and rs1042522).

## Results

**Fig 1:**
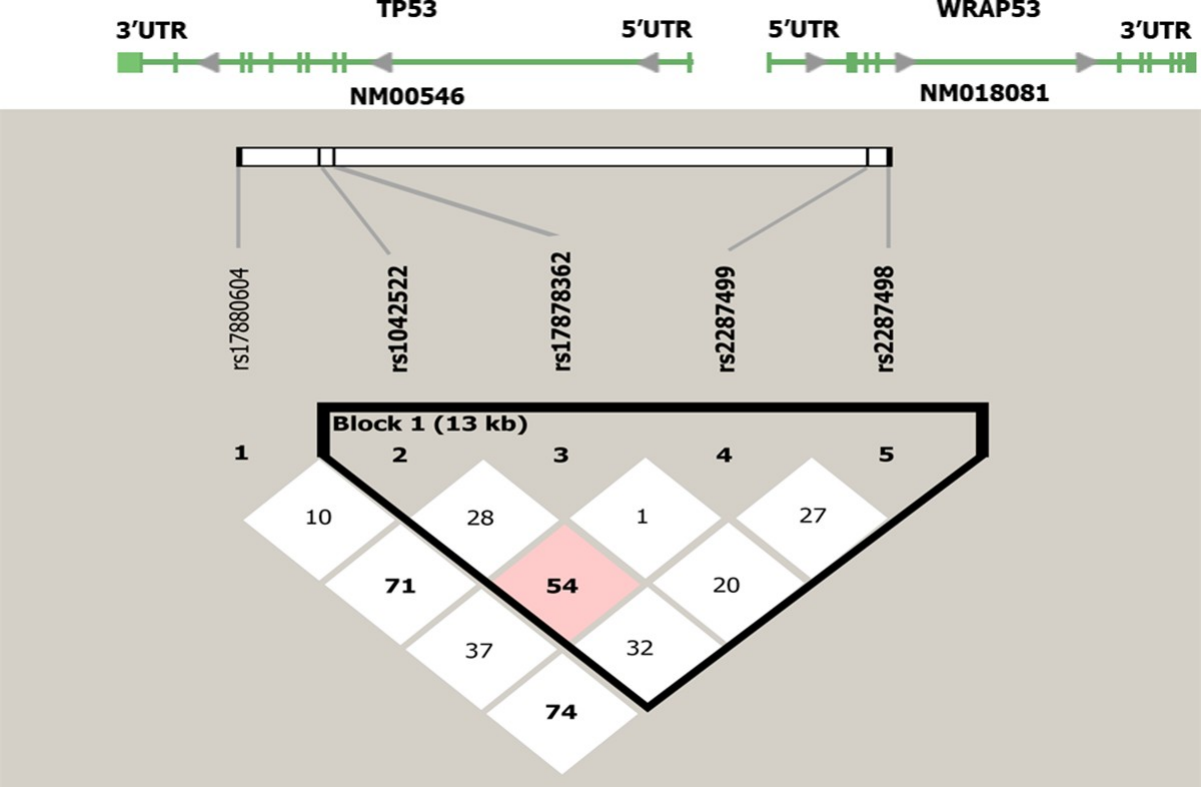
The schematic of haplotype block- formed by rs1042522, rs17878362, rs2287499, and rs2287498 SNPs in *TP53*-*WRAP53*. NM_00546 and NM_018081 are the mRNA reference sequence numbers of the TP53 and WRAP53 genes, respectively. The 5−UTR is the 5− untranslated region of the *TP53* gene. Solid squares indicate exons. The SNPs were represented in haplotype block sequentially from rs1042522, rs17878362, rs2287499, and rs2287498 (left to right).

To verify the Hardy-Weinberg equilibrium, P-value was set at 0.001, which led to the exclusion of rs17880604 from the 5 markers selected. Therefore, four of five SNPs (rs1042522, rs17878362, rs2287499, and rs2287498) were included in the analysis at the *TP53*-*WRAP53* locus as haplotype blocks that were constructed with Haploview v4.2, after which a linkage disequilibrium plot was generated (Fig 1).

As shown in this figure, there was no significant LD between any pairwise haplotypes. The highest LD was 54 and manifested between rs1042522 (C allele) of the *TP53* gene and rs2287499 (G) of the *WRAP53* gene, indicating a moderate linkage disequilibrium. The results showed that GA_1_CC was the most frequent haplotype with 0.297% and the rarest haplotypes were GA_2_CT with 0.014% frequency in both case and control samples. The GA_1_CC and CA_1_GC haplotypes were significantly associated with breast cancer susceptibility (P <0.05). The CA_1_GC haplotype was strongly identified as a breast cancer risk factor, and the GA_1_CC haplotype was assumed to be a protective factor against breast cancer risk. Subsequently, the permutation tests on the formed *TP53*-*WRAP53* haplotypes confirmed the hypothesized role of the above-mentioned haplotypes in breast cancer susceptibility (Table 1).

**Table 1:** Analysis of haplotypes formed by a combination of the four SNPs.

Haplotype^a^	Frequency (%)	Case, Control freq	*P*-value	Permutation *P*-value
GA_1_CC	0.297	0.104, 0.476	2.7926E-18	0.0000E0
CA_1_GC	0.183	0.323, 0.054	9.6397E-14	0.0000E0
CA_1_ CC	0.144	0.173, 0.116	0.0805	
CA_2_ CC	0.087	0.038, 0.132	4.0E-4	0.0010
CA_1_ GT	0.064	0.065, 0.062	0.9026	
CA_2_ GC	0.056	0.094, 0.022	9.0E-4	0.0030
GA_1_ GC	0.049	0.057, 0.041	0.4147	
GA_2_ CC	0.030	0.033, 0.028	0.7196	
GA_2_ GC	0.025	0.041, 0.009	0.029	0.1480
GA_1_ CT	0.023	0.016, 0.029	0.3625	
CA_1_ CT	0.019	0.024, 0.015	0.4759	
GA_2_ CT	0.014	0.024, 0.004	0.0701	

^a^ A_1_ and A_2_ alleles represent the 16 bp deletion and insertion within the intron 3, respectively.

Additionally, an analysis of haplotypes formed by the three markers (all combinations of the three markers formed by excluding one at a time) was performed. P-values were consistent with the aforementioned statistically significant P-values resulting from the haplotypes analysis with four markers (Table 2), suggesting that these markers in haplotype blocks formed by three or four markers are closely related to breast cancer susceptibility.

**Table 2:** Analysis of haplotypes formed by a combination of the three SNPs.

Haplotype	Frequency (%)	Case, Control freq	*P*-value	Permutation *P*-value
**Haplotype**^1^^,^ ^a^				
A_1_CC	0.438	0.269, 0.593	2.9041E-12	0.0000E0
A_1_GC	0.238	0.385, 0.102	1.3183E-12	0.0000E0
A_2_CC	0.120	0.082, 0.156	0.015	0.0670
A_2_GC	0.075	0.128, 0.027	4.3035E-5	0.0000E0
**Haplotype**^2^				
GCC	0.329	0.143, 0.500	4.3938E-16	0.0000E0
CGC	0.241	0.417, 0.079	3.2757E-17	0.0000E0
**Haplotype**^3^				
GA_2_ C	0.345	0.166, 0.510	1.0178E-14	0.0000E0
CA_1_ C	0.325	0.486, 0.176	1.4116E-12	0.0000E0
**Haplotype**^4^				
GA_1_C	0.320	0.122, 0.502	2.8545E-18	0.0000E0
CA_1_G	0.244	0.387, 0.111	6.223E-12	0.0000E0
CA_2_C	0.087	0.042, 0.130	9.0E-4	0.0070
CA_2_G	0.063	0.099, 0.029	0.0023	0.0160

^a^ A_1_ and A_2_ alleles represent the 16 bp deletion and 16 bp insertion within the intron 3, respectively.

^1^ The haplotype formed by rs17878362, rs2287499 and rs2287498.

^2^ The haplotype formed by rs1042522, rs2287499 and rs2287498.

^3^ The haplotype formed by rs1042522, rs17878362 and rs2287498.

^4^ The haplotype formed by rs1042522, rs17878362 and rs2287499.

Three of five SNPs (rs2287499, rs1042522, and rs2287498) were used for *in silico* analysis, but the other two (e.g., rs17880604 and rs17878362) were excluded due to their intronic positions. The *in silico* analysis results are shown in Table 3 such that RNAsnp analysis showed a significant alteration in secondary RNA structure of *WRAP53* due to rs2287499 polymorphism.

**Table 3:** *In silico* analysis of the studied SNPs.

Server	Scores			Prediction		
	rs2287499	rs1042522	rs2287498	rs2287499	rs1042522	rs2287498
**SIFT**	0.36	-0.23		neutral	neutral	-
**Polyphen2**	100	100	100	healthy	healthy	healthy
**Fathmm**	0.68	-5.45		tolerated	damaging	-
**RNAsnp**	*P*-value = 0.084	*P*-value = 0.2426	*P*-value = 0.9693	significant	Not significant	Not significant
**SNP&GO**	RI = 9	RI = 9		neutral	neutral	-

**Fig 2:**
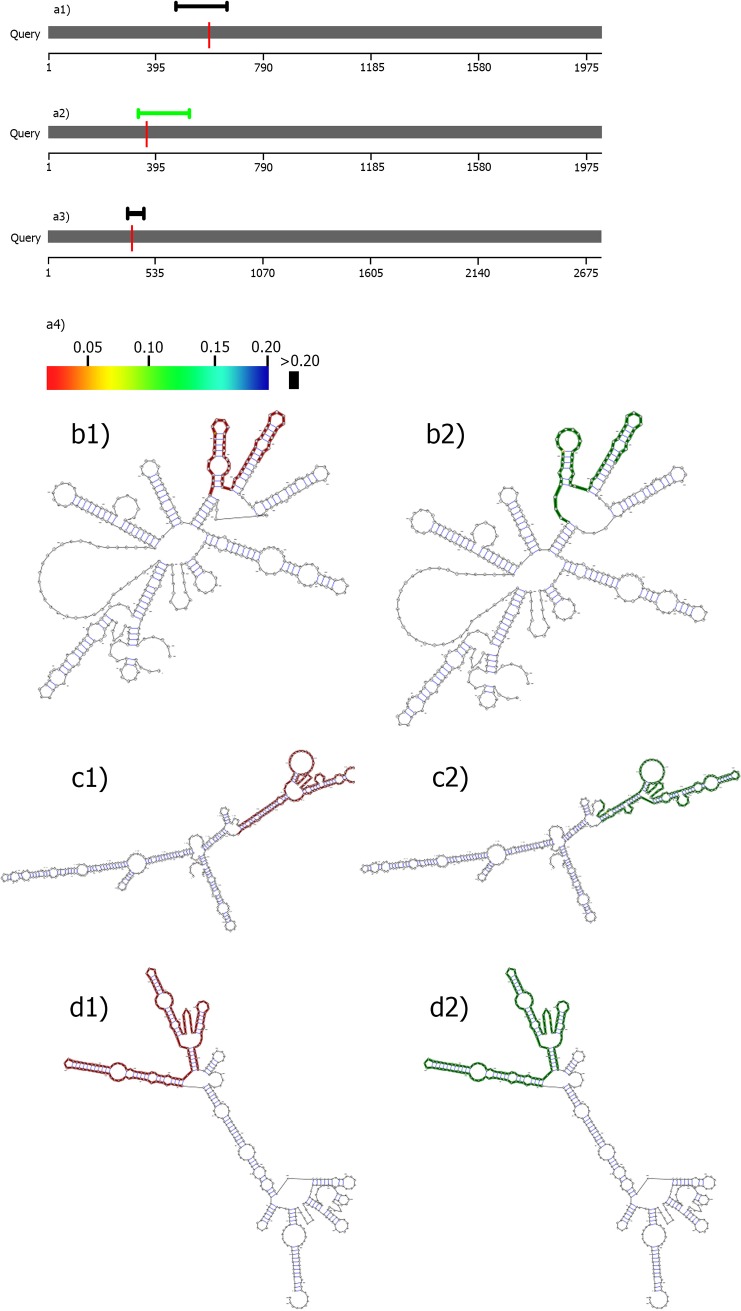
RNAsnp analysis of rs2287499, rs1042522 and rs2287498 SNPs. Local regions for a1) R72P substitution in *TP53*, a2) R68G substitution in *WRAP53*, a3) F150F substitution in *WRAP53*. a4) black lines demonstrate insignificant alteration (P >0.02) and other colors are demonstration of significant changes. Secondary RNA structure of R72P substitution in b1) mutation and b2) wild-type. Secondary RNA structure of R68G substitution in c1) mutation and c2) wild-type. Secondary RNA structure of F150F substitution in d1) mutation and d2) wild-type.

Also, Fathmm website predicted damaging statues for rs1042522 polymorphism in p53 protein. Secondary RNA structure alterations of *TP53* and *WRAP53* were observed due to the mentioned three SNPs as shown in Fig 2.

## Discussion

Polymorphisms and haplotypes in the *TP53* and *WRAP53* genes may affect their products, thereby causing cancer vulnerability, tumor invasiveness and prognosis, or cancer therapy response [20,29,30]. Our results demonstrated an increased risk of breast cancer in one out of 12 possible combinations of polymorphisms (the CA_1_GC haplotype block)(). Additionally, one of the haplotypes (GA_1_CC) was assumed to be a protective risk factor. These imply the essential roles of the haplotypes at the *TP53*-*WRAP53* locus, possibly due to the crucial role of p53 in various cell functions and due to *WRAP53* being a regulator of p53 or a vital RNP biogenesis mediator.

Also, with moderate D′ values between rs1042522 and rs2287499 SNPs (Fig 1), it is obvious that there is only a small chance that these markers will be inherited together from the same ancestor, suggesting heterogeneity in our studied SNPs in an Iranian-Azeri population. A number of researchers have investigated the haplotype linkage disequilibrium in the *TP53*-*WRAP53* locus region. Naccarati et al. analyzed the *TP53* gene haplotype consisting of four SNPs, including rs17878362, rs1042522, rs12947788, and rs17884306 in a Czech Republic population. They showed a haplotype association (A_1_CCG) with cancer risk [31]; nonetheless, they proved that the A_2_GCG haplotype is associated with a significan decrease in the risk of breast cancer (P = 0.0001) [32]. Xingqun et al. investigated eight SNPs in the *TP53*-*WRAP53* locus as a haplotype in Toronto and Portuguese populations for schizophrenia susceptibility. They showed a significant linkage disequilibrium between rs17878362 and rs2287499 and between rs2287499 and rs2287498 polymorphisms with a D′ value of 100 in both Toronto and Portuguese populations, respectively [33]. Our results are in contrast to the results of this study because we did not obtain a significant LD between haplotypes formed by the four investigated SNPs. The pairwise haplotype analysis between *TP53* Arg72Pro and *WRAP53* rs2287499 in HapMap Caucasians did not show a strong LD (D′ = 48) [34], which was in agreement with our results (D′ = 54). Besides, our previous pairwise analysis on rs2287499 and rs1042522 did not show a strong LD (D′ = 32), but we found a significant association between the GC haplotype with breast cancer risk (P = 0.024) [35]. Our data were consistent with a study by Buyru et al., who analyzed three SNPs (rs17878362, rs1042522, and rs17880604) in two polymorphism combinations (+16 bp GG and –16 bp GG) since this haplotype showed an association with an increase in the risk of breast cancer in a Turkish population [36]. *TP53* haplotype comprising of rs17878362, rs1042522 and rs186837503 for the incidence risk of post-angioplasty restenosis was evaluated by Y. L. Zee et al. They demonstrated two polymorphism combinations (–16 bp CG and +16 bp CA) as a protective haplotype [37] which was in accordance with the Hao et al. [38] studies relating to breast cancer risk (investigating rs17878362 and rs1042522), but our study did not confirm it. These conflicting results may be explained by allele frequency differences between ethnic groups or be due to different sample sizes.

We also conducted an *in silico* analysis which revealed the impact of rs2287499 and rs1042522 polymorphisms on *WRAP53* secondary RNA structure by a probable destabilizing effect and on cancer risk presumably by affecting the p53 protein function, respectively.

## Conclusion

In conclusion, we found that the CA_1_GC and GA_1_CC haplotypes confer an increased risk of breast cancer and a protective role in breast cancer susceptibility, respectively. Therefore, they may be useful as molecular prognostic markers for breast cancer, suggesting that genetic background based on certain haplotypes in *TP53*-*WRAP53* genes may play an important role in breast cancer susceptibility. In addition, LD analysis between four studied polymorphisms indicates heterogeneity of our studied population and provides a presumption that these markers are not linked together. Furthermore, *in silico* analysis predicted the alterations in *TP53* and *WRAP53* products in presence of rs1042522 and rs2287499, respectively.

The important functions of *WRAP53* and the different polymorphisms within it for regulating p53 are emerging. To the best of our knowledge this is the first study to describe the haplotypes of the *TP53*-*WRAP53* locus. However, more studies are needed to investigate more haplotype blocks within this locus in order to elucidate the complex regulating pathways and to discover cancer-associated markers with possible prognostic utility.

## Supporting information

S1 Data Clinicopathologic data of all the studied cases. (XLSX)

S2 Data Genotypes for all the five SNPs in the studied population related to Tables 1 and 2. (XLSX)
